# The Potential of Bioactive Fish Collagen Oligopeptides against Hydrogen Peroxide-Induced NIH/3T3 and HUVEC Damage: The Involvement of the Mitochondria

**DOI:** 10.3390/nu16071004

**Published:** 2024-03-29

**Authors:** Na Zhu, Rui Liu, Meihong Xu, Yong Li

**Affiliations:** 1 Department of Nutrition and Food Hygiene, School of Public Health, Peking University, Beijing 100191, China; summer920503@163.com (N.Z.); liuruipku@163.com (R.L.); xumeihong@bjmu.edu.cn (M.X.); 2 Department of Nutrition and Food Hygiene, College of Public Health, Inner Mongolia Medical University, Hohhot 010059, China; 3 Institute of Advanced Clinical Medicine, Peking University, Beijing 100191, China; 4 Beijing Key Laboratory of Toxicological Research and Risk Assessment for Food Safety, Peking University, Beijing 100191, China

**Keywords:** fish collagen oligopeptides, hydrogen peroxide, inflammation, mitochondria

## Abstract

Extensive in vivo investigations have demonstrated the antioxidant properties of fish collagen oligopeptides (FCOPs). One of the main causes of aging and chronic non-communicable diseases is oxidative stress. Therefore, FCOPs have a broad range of applications in illness prevention and delaying aging from the standpoint of the “food is medicine” theory. However, the mechanisms that underpin the antioxidant activity of FCOPs are not completely understood. The specific objective of this essay was to investigate the antioxidant effect of FCOPs and its possible mechanism at the cellular level. Mouse embryonic fibroblasts NIH/3T3 and human vein endothelial cells (HUVECs) were exposed to 200 µM hydrogen peroxide containing different concentrations of FCOPs for 4 h and were supplemented with different concentrations of FCOPs for 24 h. Normal growth medium without FCOPs was applied for control cells. An array of assays was used to evaluate the implications of FCOPs on cellular oxidative stress status, cellular homeostasis, inflammatory levels, and mitochondrial function. We found that FCOPs exerted a protective effect by inhibiting reactive oxygen species (ROS) production, enhancing superoxide dismutase (SOD) and endothelial nitric oxide synthase (eNOS) activities and cell viability, inhibiting cell cycle arrest in the G1 phase, suppressing interleukin-1β (IL-1β), IL-6, matrix metalloproteinase-3 (MMP-3) and intercellular adhesion molecule-1(ICAM-1) secretion, downregulating nuclear factor-kappa B (NF-κB) activity, protecting mitochondrial membrane potential, and increasing ATP synthesis and NAD^+^ activities in both cells. FCOPs had a stronger antioxidant impact on NIH/3T3 than on HUVECs, simultaneously increasing glutathione peroxidase (GSH-Px) activity and decreasing malondialdehyde (MDA) content in NIH/3T3. These findings indicate that FCOPs have antioxidant effects on different tissue cells damaged by oxidative stress. FCOPs were therefore found to promote cellular homeostasis, inhibit inflammation, and protect mitochondria. Meanwhile, better health outcomes will be achieved by thoroughly investigating the effective dose and intervention time of FCOPs, as the absorption efficiency of FCOPs varies in different tissue cells.

## 1. Introduction

Redox homeostasis is central to life. Redox processes are involved in almost all fundamental processes, from bioenergy to metabolism and life functions [1]. During this process, disturbances in the balance between oxidation and antioxidation lead to potential damage, known as oxidative stress [2]. Research has confirmed that oxidative stress-induced damage is the pathophysiological basis of various diseases [3]. The primary consequence of oxidative stress is damage to biological macromolecules, including nucleic acids, proteins, and fats, leading to oxidation, hydrolysis, and methylation of DNA, as well as the accumulation of unfolded proteins and lipid oxidation products [1,4]. The onset of multiple chronic diseases such as diabetes and its complications, hypertension, atherosclerosis, tumors, kidney damage, and neurodegenerative disease are significantly influenced by these impairments [5,6,7,8]. The aging process of the organism is also a major contributor to many chronic disorders [9]. The theory of aging is centered on the proposition that age-related functional deficits are caused by the accumulation of oxidative stress-induced injury [10]. Thereby, Redox homeostasis is “the golden mean of healthy living” [11]. Mitochondria are the primary site of redox metabolism and are the organelle with the highest content of free radicals. They act as the cellular powerhouse and synthesize ATP primarily through the tricarboxylic acid cycle and the electron transport chain. Normally, this is an efficient process with minimal electron leakage. However, pathological circumstances result in mitochondrial dysfunction and increased electron leakage, which raises the generation of O_2_^−^. With superoxide dismutase (SOD), O_2_^−^ is changed to H_2_O_2_, which passes through the mitochondrial membrane into the cytoplasm. At the same time, exogenous free radicals can also attack mitochondrial membrane potential, respiratory chain complexes, and mitochondrial DNA, which can lead to mitochondrial dysfunction and exacerbate the generation of free radicals [9,12]. Mitochondrial dysfunction is a potential trigger for inflammation and cell death. A growing body of research shows that mitochondrial dysfunction is strongly associated with metabolic diseases, neurological disorders, cardiovascular diseases (CVDs), tumors, and muscle diseases [13].

Food is medicine, and the global epidemic of chronic diseases has prompted the integration of specific foods and nutritional interventions with healthcare systems [14,15]. Exploring the additional health benefits of food and its active components is of profound significance. Bioactive peptides are typical representatives in this research field. The current investigation concentrated on the advantages of bioactive peptides isolated from fish collagen. Fish collagen oligopeptides (FCOPs) are small molecule oligopeptides derived from fish meat or byproducts such as fish skin, fish scales, fish bones, and offal fish collagen, which is decomposed by proteolytic and other technologies. FCOPs are easily absorbed and utilized by the body, providing essential nutrients for growth and development, participating in the material and energy metabolism of the body, and exerting various biological activities [16]. Over the years, our group has extensively studied the potential health effects of FCOPs and confirmed that FCOPs have an abundance of biological activities such as antioxidant activity [17], anti-alcohol toxicity [18], immunomodulation [19], regulation of glucose metabolism [20], promotion of wound healing [21], and extension of lifespan [22]. We hypothesized that the fundamental mechanism behind these health effects is their antioxidative characteristic. This assumption is based on our findings that FCOPs significantly enhanced liver or serum SOD and glutathione peroxidase (GSH-Px) activities and inhibited malondialdehyde (MDA) production in ^60^Coγ-radiation injured mice [17], alcohol fatty liver disease rats [18], streptozotocin-induced diabetic rats [20], and lifelong feeding aged rats [22]. To support and extend the findings of previous in vivo research on mice, we selected mouse embryonic fibroblasts NIH/3T3, which are commonly employed in the cell senescence and oxidative stress model [23,24], to evaluate the protective effect of FCOPs on oxidative stress-induced damaged cells and its possible biological mechanism. Considering that the role of antioxidants may vary in different species and different tissues, we once more selected HUVECs, which are frequently utilized in the model of CVDs, since endothelial dysfunction caused by various factors, including oxidative stress, is the initiating factor of CVDs [25,26]. Given that CVDs are the most fatal chronic non-communicable illness, research into the protective role of FCOPs against oxidatively damaged HUVECs is crucial.

## 2. Materials and Methods

### 2.1. FCOPs

The FCOPs were obtained from Anhui Shengmeinuo Biotechnology Co., Ltd. (Huaibei, China). They were derived from Tilapia (*Oreochromis mossambicus*) scales by the method of enzymatic hydrolysis. The FCOPs consisted of a blend of active peptides with molecular weights under 1000 Dalton. Among them, the peptides with molecular weights between 180–500 Da, less than 180 Da, and between 500 and 1000 Da accounted for 56.06%, 9.97%, and 26.67%, respectively. Detailed amino acid information for the FCOPs used in this study is shown in Table 1.

### 2.2. Study Design

NIH/3T3 and HUVECs were obtained from the ATCC (Manassas, VA, USA). Cells were plated in Dulbecco’s Modification of Eagle’s Medium (Corning, Shanghai, China), supplemented with 1% antibiotic-antimitotic (Gibco, Grand Island, NY, USA) and 10% fetal bovine serum (Gibco, Grand Island, NY, USA) under 5% CO_2_ at 37 °C.

A total of five treatment groups were involved in each cell line: the vehicle control group, the H_2_O_2_ control group, and the three doses of FCOPs groups. The vehicle control group was supplemented with a growth medium. The H_2_O_2_ control group was incubated for 4 h in a growth medium containing 200 µM of H_2_O_2_ and then changed to a normal growth medium for 24 h. The FCOP intervention groups were cultured in a growth medium containing 200 µM of H_2_O_2_ and 25, 50, and 100 µg/mL of FCOPs, and 4 h later, the cells were seeded for 24 h in an H_2_O_2_ free growth medium containing 25, 50, and 100 µg/mL of FCOPs. After different treatments, the cells were gathered for further investigation. Given that FCOPs are food-derived compounds, they work more successfully at avoiding harm or illness than at healing it. Therefore, the cells were incubated with H_2_O_2_ and FCOPs concurrently. Furthermore, as it has been reported that low doses of H_2_O_2_ activate compensatory responses, the cells were exposed to higher doses of H_2_O_2_ over a short period to achieve an acute injury model.

### 2.3. Cell Viability

The cell-counting kit-8 test (KeyGEN, Nanjing, China) was used to measure cell viability, in accordance with the manufacturer’s instructions. In 96-well plates, roughly 1 × 10^4^ cells/well were introduced. Following the prescribed treatment, 10 μL of CCK-8 solution was added to each well and maintained for 1–4 h at 37 °C. Using a microplate reader (BMG FLUOstar Omega, Offenburg, Germany), the absorbance of every well was determined at 450 nm.

### 2.4. Flow Cytometry

Cells were inoculated in 6-well plates and processed according to the protocol described in Section 2.2. Cells were digested with trypsin and washed twice with pre-chilled phosphate-buffered saline (PBS), before being maintained at 4 °C overnight with 75% ethanol. Cells were washed three times with pre-chilled PBS and maintained at 37 °C for 30 min with propidium staining and RNase A (Beyotime, Shanghai, China), and a Flow Cytometer (Beckman Coulter, Brea, CA, USA) was used to analyze the cell cycle. To evaluate the reactive oxygen species (ROS) production, cells were digested and washed once in pre-chilled PBS, then maintained at 37 °C for 20 min with 10 μM of 2,7-dichlorofluorescein diacetate (Beyotime, Shanghai, China). The cells were washed three times and the ROS level was tested with a flow cytometer. Cells were digested and maintained at 37 °C for 20 min in the dark with 1 mL growth medium and 1 mL 1 × JC-1 staining solution (Beyotime, Shanghai, China). Then, the cells were washed with pre-chilled PBS and cultured in 1× JC-1 staining buffer. The mitochondrial membrane potential (∆Ψm) of the cells was detected utilizing flow cytometry.

### 2.5. Biochemical Analysis

Cells (2 × 10^5^/well) were treated according to the experimental procedure mentioned in Section 2.2 and then washed with pre-chilled PBS. Then, 200 μL NAD^+^ and NADH extracting solution was added into each well to achieve cell lysis. Centrifugation was performed at 12,000 rpm for 10 min to extract the supernatant, and the levels of NAD^+^ and NADH were detected in each group using the WST-8 method (Beyotime, Shanghai, China). The contents of malondialdehyde (MDA), glutathione peroxidase (GSH-Px), superoxide dismutase (SOD), telomerase (TE), interleukin-6 (IL-6), IL-1β, matrix metalloproteinase-3 (MMP-3), and intercellular cell adhesion molecule-1 (ICAM-1) in the supernatant of each group were evaluated according to the commercial kits protocol. The MDA, GSH-Px, and SOD kits were purchased from Nanjing Jiancheng (Nanjing, China). The IL-6 and IL-1β kits were obtained from Invitrogen (Waltham, MA, USA). The MMP-3 and ICAM-1kits were purchased from Multisciences (Hangzhou, China). The TE activity kit was purchased from FEIYA (Nantong, China).

### 2.6. Western Blot Analysis

The cells were rinsed three times in PBS. Then, 200 uL of RIPA Lysis Buffer (Biosharp, Hefei, China) was added to each well to fully lyse the cells. The cell lysis solutions were centrifugated at 12,000 rpm for 5 min at 4 °C. The sampling buffer was added into the supernatant and the protein was boiled in a 100 °C water for 7 min until denaturation, before being naturally cooled to room temperature. The protein concentrations were then determined according to the instructions of the BCA Protein Concentration Assay Kit (Thermo Scientific, Waltham, MA, USA). Aliquots of protein samples were separated by SDS-PAGE electrophoresis. The separated protein samples were transferred to PVDF membranes by wet electro-transfer, and the current and time of membrane transfer were determined according to the size of the protein molecules to be measured. The membrane was placed in a sealing solution and gently shaken for 2 h at room temperature, and the membrane was washed with TBST four times after the sealing had been set. The corresponding primary antibody was diluted to the appropriate concentration and hybridized at 4 °C overnight. The membrane was hybridized with horseradish peroxidase-labeled secondary antibody for 2 h at room temperature. Luminescent solution was added to the membrane and a gel imaging analyzer was utilized. Image-Pro Plus (Media Cybernetics, Rockville, MD, USA) was used for quantitative protein analysis. Primary antibodies γ-H2A.X, *p*-NF-κB, NF-κB, PGC-1α, and β-actin were purchased from Abcam (Cambridge, MA, USA). SIRT1 and AMPKα antibodies were purchased from CST (Danvers, MA, USA). We analyzed the protective effects of different doses of FCOPs and found that FCOPs25 had the most prominent overall effect on all the indicators evaluated in the present study. Therefore, we selected this group for protein analysis.

### 2.7. Statistical Analysis

Version 24 of SPSS (SPSS Inc., Chicago, IL, USA) was used to conduct statistical analyses. The one-way analysis of variance (ANOVA) test was used to analyze the data, which are expressed as mean ± standard deviation (SD). LSD test (with equal variances assumed) or Dunnett’s T3 test (with equal variances not assumed) were used for comparisons between three or more groups. The test level was *p* < 0.05.

## 3. Results

### 3.1. FCOP Enhanced Antioxidant Activities in H_2_O_2_-Treated NIH/3T3 and HUVECs

**Figure 1:**
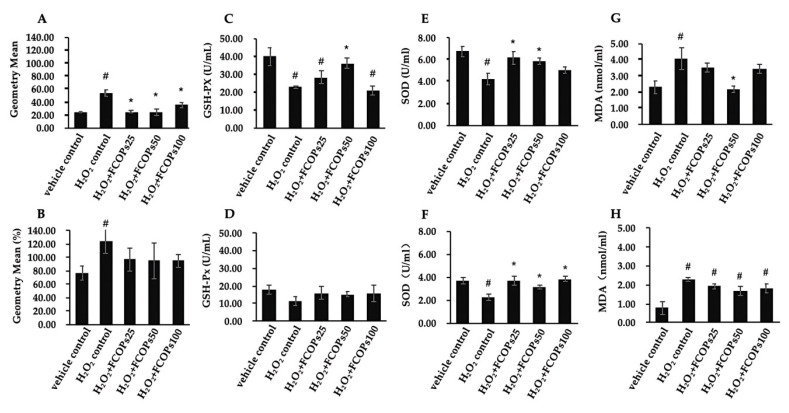
(**A**) ROS production in NIH/3T3. (**B**) ROS production in HUVECs. (**C**) GSH-Px activities in NIH/3T3. (**D**) GSH-Px contents in HUVECS. (**E**) SOD levels in NIH/3T3. (**F**) SOD contents in HUVECs. (**G**) MDA contents in NIH/3T3. (**H**) MDA contents in HUVECs. Values are shown as mean ± S.D. (*n* = 3 per group). # *p* < 0.05 vs. vehicle control, * *p* < 0.05 vs. H_2_O_2_ control.

Relative to the H_2_O_2_ control group, different dosages of FCOPs administration significantly reduced the ROS production in NIH/3T3 (*p* < 0.05) (Figure 1A). The inhibitory effect of FCOPs on ROS production was able to control ROS at normal levels compared with the vehicle control group in HUVECs (*p* > 0.05) (Figure 1B). Notably, 50 µg/mL FCOP administration statistically boosted the GSH-Px activity compared with the H_2_O_2_ control group in NIH/3T3 (*p* < 0.05) (Figure 1C). The GSH-Px activities did not change among all groups in HUVECs (*p* > 0.05) (Figure 1D). Compared with the H_2_O_2_ control, 25 and 50 µg/mL FCOP supplementation dramatically increased the SOD activities in NIH/3T3, while three dosages of FCOPs significantly enhanced the SOD activities in HUVECs (*p* < 0.05) (Figure 1E,F). Compared with the H_2_O_2_ control, 50 µg/mL FCOPs administration significantly inhibited MDA content in NIH/3T3 (*p* < 0.05) (Figure 1G). FCOPs did not affect the MDA content in HUVECs compared with the H_2_O_2_ control (*p* > 0.05) (Figure 1H).

### 3.2. FCOPs Promote Homeostasis in H_2_O_2_-Treated NIH/3T3 and HUVECs

**Figure 2:**
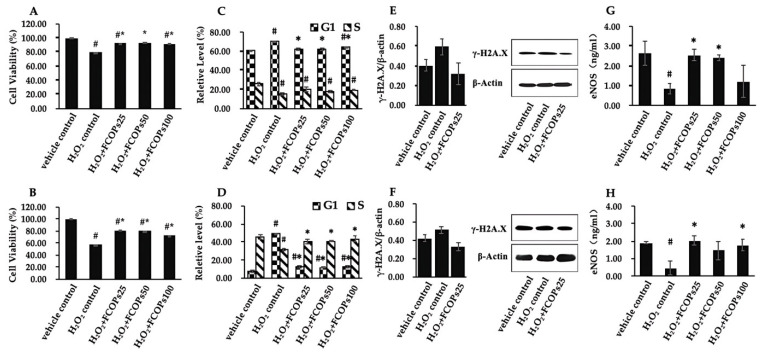
(**A**) Cell viability in NIH/3T3. (**B**) Cell viability in HUVECs. (**C**) Cell cycle distribution in NIH/3T3. (**D**) Cell cycle distribution in HUVECs. (**E**) The γ-H2A.X expression in NIH/3T3. (**F**) The γ-H2A.X expression in HUVECs. (**G**) eNOS activities in NIH/3T3. (**H**) eNOS activities in HUVECs. Values are shown as mean ± S.D. (*n* = 3 per group). # *p* < 0.05 vs. vehicle control, * *p* < 0.05 vs. H_2_O_2_ control.

Relative to the H_2_O_2_ control, three dosages of FCOP significantly increased cell viability, both in NIH/3T3 and HUVECs (*p* < 0.05) (Figure 2A,B). Three dosages of FCOP significantly inhibited G1 phase cell cycle blocking, both in H_2_O_2_-treated NIH/3T3 and HUVECs (*p* < 0.05), while those also significantly increased the cell proportion in the S phase in HUVECs (*p* < 0.05) (Figure 2C,D). Compared with the vehicle control, H_2_O_2_ treatment tended to increase the γ-H2A.X content, while FCOP supplementation tended to decrease the γ-H2A.X expression, both in NIH/3T3 and HUVECs (*p* > 0.05) (Figure 2E,F). With respect to the H_2_O_2_ control, 25 and 50 µg/mL FCOP supplementation significantly enhanced the eNOS activities in NIH/3T3, while 25 and 100 µg/mL FCOP administration significantly enhanced eNOS activities in HUVECs (*p* < 0.05) (Figure 2G,H).

### 3.3. FCOPs Suppressed Inflammation in H_2_O_2_-Treated NIH/3T3 and HUVECs

**Figure 3:**
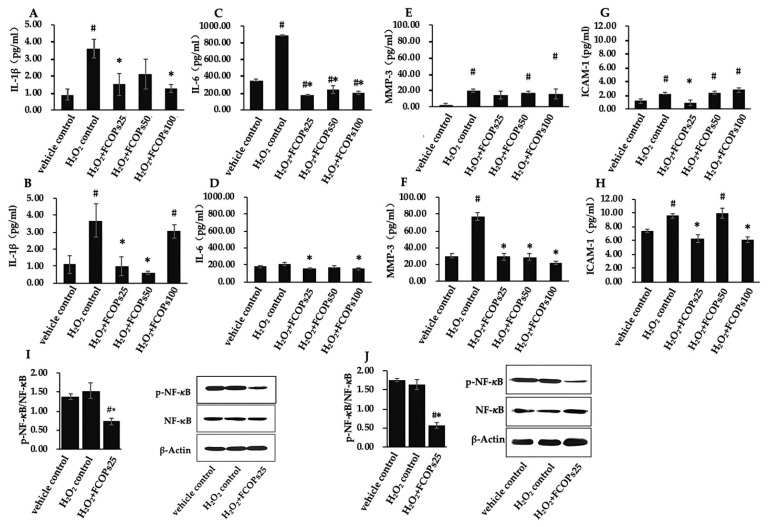
(**A**) IL-1β secretion in NIH/3T3. (**B**) IL-1β secretion in HUVECs. (**C**) IL-6 secretion in NIH/3T3. (**D**) IL-6 secretion in HUVECs. (**E**) MMP-3 secretion in NIH/3T3. (**F**) MMP-3 secretion in HUVECs. (**G**) ICAM-1 secretion in NIH/3T3. (**H**) ICAM-1 secretion in HUVECs. (**I**) *p*-NF-κB/NF-κB in NIH/3T3. (**J**) *p*-NF-κB/NF-κB in HUVECs. Values are shown as mean ± S.D. (*n* = 3 per group). # *p* < 0.05 vs. vehicle control, * *p* < 0.05 vs. H_2_O_2_ control.

Compared with the H_2_O_2_ control group, 25 and 100 µg/mL FCOP administration significantly inhibited IL-1β secretion in NIH/3T3, while 25 and 50 µg/mL FCOP significantly inhibited IL-1β secretion in HUVECs (*p* < 0.05) (Figure 3A,B). Compared with the H_2_O_2_ control, all concentrations of FCOP administration statistically inhibited IL-6 secretion in NIH/3T3, 25 and 100 µg/mL FCOP significantly inhibited IL-6 secretion in HUVECs (*p* < 0.05) (Figure 3C,D). Compared with the H_2_O_2_ control, FCOP administration did not affect MMP-3 secretion in NIH/3T3 (*p* > 0.05) (Figure 3E), and three dosages of FCOPs statistically suppressed MMP-3 production in HUVECs (*p* < 0.05) (Figure 3F). Additionally, 25 µg/mL administration significantly inhibited ICAM-1 secretion compared with the H_2_O_2_ group in NIH/3T3, and 25 and 100 µg/mL FCOP administration significantly inhibited ICAM-1 secretion compared with the H_2_O_2_ group in HUVECs (*p* < 0.05) (Figure 3G,H). Furthermore, 25 g/mL FCOP treatment significantly reduced NF-κB activation compared with the vehicle control and H_2_O_2_ control group, both in NIH/3T3 and HUVECs (*p* < 0.05) (Figure 3I,J).

### 3.4. FCOPs Influenced Mitochondrial Function and Biogenesis in H_2_O_2_-Treated NIH/3T3 and HUVECs

**Figure 4:**
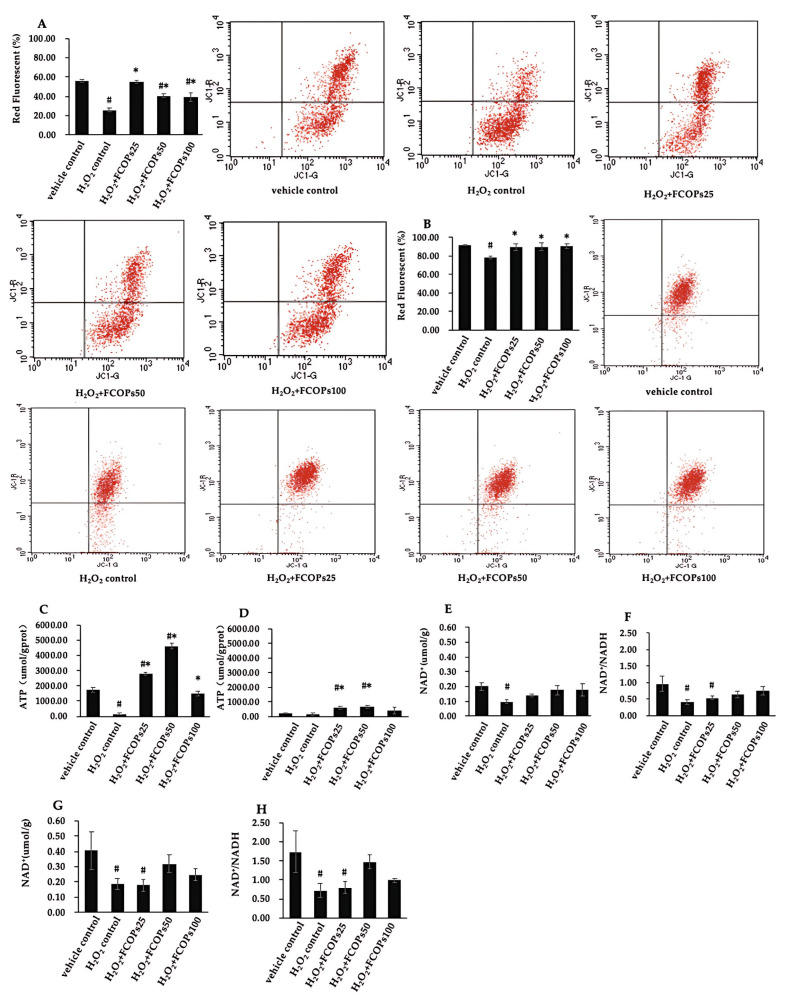
(**A**) The mitochondrial membrane potential in NIH/3T3. (**B**) The mitochondrial membrane potential in HUVECs (**C**) ATP production in NIH/3T3. (**D**) ATP production in HUVECs. (**E**) NAD^+^ activities in NIH/3T3. (**F**) NAD^+^/NADH in NIH/3T3. (**G**) NAD^+^ activities in HUVECs. (**H**) NAD^+^/NADH in HUVECs. Values are shown as mean ± S.D. (*n* = 3 per group). # *p* < 0.05 vs. vehicle control, * *p* < 0.05 vs. H_2_O_2_ control.

Relative to the H_2_O_2_ control, FCOP treatment dramatically enhanced mitochondrial membrane potential, both in NIH/3T3 and HUVECs (*p* < 0.05) (Figure 4A,B). Three dosages of FCOPs significantly increased ATP production with respect to the H_2_O_2_ control in NIH/3T3 (*p* < 0.05) (Figure 4C). In HUVECs, 25 and 50 µg/mL FCOP exposure significantly increased ATP production compared to the two control groups (*p* < 0.05) (Figure 4D). Compared with the vehicle control, H_2_O_2_ treatment significantly reduced NAD^+^ activity and NAD^+^/NADH both in NIH/3T3 and HUVECs (*p* < 0.05) (Figure 4E–H), while three dosages of FCOPs returned the NAD^+^ activities to the normal level in NIH/3T3. Additionally, 50 and 100 µg/mL FCOP supplementation returned the NAD^+^ activities and NAD^+^/NADH to the normal levels both in NIH/3T3 and HUVECs.

**Figure 5:**
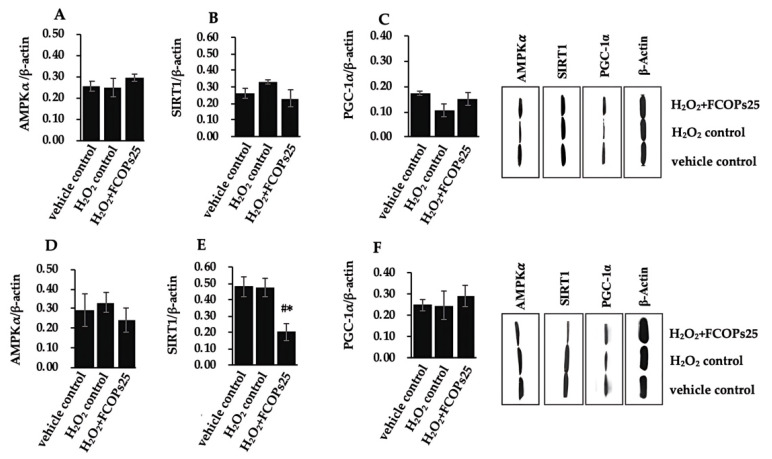
(**A**) AMPKα expression in NIH/3T3. (**B**) SIRT1 expression in NIH/3T3. (**C**) PGC-1α expression in NIH/3T3. (**D**) AMPKα expression in HUVECs. (**E**) SIRT1 expression in HUVECs. (**F**) PGC-1α expression in HUVECs. Values are shown as mean ± S.D. (*n* = 3 per group). # *p* < 0.05 vs. vehicle control, * *p* < 0.05 vs. H_2_O_2_ control.

We further evaluated the effects of FCOPs on the AMPK/NAD^+^/SIRT1/PGC-1α signaling pathway. In NIH/3T3, the AMPKα, SIRT1, and PGC-1α expression did not statistically differ among the groups (*p* > 0.05) (Figure 5A–C), while SIRT1 expression tended to up-modulate in the H_2_O_2_ control and tended to down-modulate in the FCOPs administration group in NIH/3T3. In contrast, the PGC-1α expression tended to decrease in the H_2_O_2_ control and tended to upregulate in the FCOP administration group in NIH/3T3. Multiple treatments did not affect the AMPKα expression in HUVECs (*p* > 0.05) (Figure 5D). Additionally, 25 µg/mL FCOP administration significantly reduced the SIRT1 expression compared to the vehicle control and H_2_O_2_ control group in HUVECs (*p* < 0.05) (Figure 5E). FCOP administration tended to upregulate PGC-1α expression with respect to the vehicle control and H_2_O_2_ control in HUVECs (*p* > 0.05) (Figure 5D).

## 4. Discussion

This study investigated the antioxidant effect of FCOPs at the cellular level and elucidated their cellular biological mechanisms. In vitro investigations on NIH/3T3 and HUVECs revealed that FCOPs have strong antioxidant effects, protecting cells by suppressing ROS production, increasing antioxidant enzyme activity, improving cell homeostasis, inhibiting inflammation, and preserving mitochondria. However, the above-mentioned protective impact of FCOPs differed significantly across the two types of cells.

We showed that FCOPs possess an excellent radical scavenging ability in NIH/3T3. Compared to NIH/3T3, FCOPs have weaker free radical scavenging ability in HUVECs but can inhibit ROS production and maintain it at normal levels in HUVECs. The following factor may be attributed to the difference in ROS generation between the two cell lines: HUVECs have a higher energy demand than NIH/3T3, which influences the activity of intracellular oxidative phosphorylation and the electron transport chain, thereby effecting ROS generation. The present study also showed that the baseline level of ROS in HUVECs was twice that of NIH/3T3. Furthermore, in NIH/3T3, FCOPs significantly increased both GSH-Px and SOD activities, resulting in a higher ability to inhibit ROS generation and scavenge them. Overall, the potentiating implication of FCOPs on antioxidant enzymes is consistent with the results of our in vivo experiments. We found that 30 days of administration of 0.450 and 0.225 g/(kg.d) of FCOPs significantly enhanced liver GSH-Px and SOD activities and reduced MDA production in 4.5 Gy (1.5 Gy/min) ^60^Coγ-rays irradiated C57BL mice [17]. Ionizing radiation (X-rays, γ-rays, etc.) can ionize water molecules of organisms. This process generates an enormous amount of free radicals, which can cause oxidative stress damage to the body [27]. Therefore, we demonstrated the antioxidant effects of FCOPs using in vivo and in vitro oxidative stress models. In addition, several studies have reported that the collagen peptides from other marine sources, including Siberian sturgeon cartilage, tilapia skin, and milkfish scales, exhibit the capacities of scavenging free radicals and enhancing antioxidant enzyme activities [28,29,30]. These findings support the hypothesis that the antioxidant effects of bioactive peptides target antioxidant enzymes; this may be due to the fact that bioactive peptides specifically complement the pool of amino acids required for the synthesis of antioxidant enzymes [31]. The present research raises the possibility that the antioxidant properties of FCOPs are linked to their free radical scavenging capacity, further elucidating the mechanism of the antioxidant effects of FCOPs. Furthermore, the antioxidant characteristics of FCOPs may be explained by their molecular weight and amino acid content. Peptides with small molecular weights and high proportions of polar amino acids are more likely to enter free radical reaction centers and exhibit stronger antioxidant activity [32]. FCOPs are oligopeptides whose molecular weights are mainly concentrated in the 180–500 DA range and whose polar amino acid proportion reaches more than 60%.

Oxidative stress and cellular homeostasis are interdependent. Cellular homeostasis can be disrupted by oxidative stress, yet cellular damage brought on by oxidative stress can be lessened when cellular homeostasis is maintained [33]. The implications of oxidative stress on cellular homeostasis are mainly manifested in DNA damage, oxidized lipid damage, protein damage, and apoptosis [34]. In this study, we show that FCOPs significantly cemented cell proliferation and inhibited G1-phase cell cycle blocking in both NIH/3T3 and HUVECs. Notably, FCOPs significantly promoted DNA synthesis (S phase) in HUVECs; however, no similar occurrence was observed in NIH/3T3. This phenomenon could have several causes. For example, HUVECs, which are located in the human blood vessel lining, are often more active and require high metabolic activity to maintain their normal function [35]. Therefore, they might utilize the basic materials for DNA synthesis more rapidly when under stress. Furthermore, given that NIH/3T3 and HUVECs come from distinct species and organs, variations in cell signaling pathways, cell surface receptors, and metabolic pathways are inevitable. Certainly, more thorough research is required to clarify the precise mechanisms underlying the various factors that can contribute to differences in the effects of FCOPs. FCOPs tended to decrease γ-H2A.X expression, the biomarker of DNA damage, in both cell types. In addition, FOCPs significantly enhanced the activity of the endothelial nitric oxide synthase, which is considered a protective factor against damage [36]. Overall, FCOPs serve to stabilize cellular homeostasis, ensuring normal cellular activity and reducing the production of damage markers. Consistent with our results, Baskaran et al. demonstrated that collagen peptides from Taiwan tilapia skin could increase the cell proportion in the G1 and S phases in different lung cells [37]. Furthermore, the DNA protective effects of collagen peptides derived from different species including fish have been investigated by several research teams [30,38].

During oxidative stress, ROS causes direct damage to cellular components and indirectly damages them by activating local inflammation. Inflammation, in turn, can activate ROS generation, creating a feedback loop that exacerbates injury [39]. This study discovered that FCOPs have significant anti-inflammatory effects in cells. The physiological levels of the inflammatory cytokines indicated in this paper, as well as their response to H_2_O_2_ and FCOPs, varied between the two types of cells. Specifically, the physiological levels of IL-6 in NIH/3T3 were slightly higher than in HUVECs. When exposed to 200 µM of H_2_O_2_, the secretion of IL-6 increased almost fourfold in NIH/3T3, but acute injury did not alter the IL-6 content in HUVECs. Meanwhile, the capacity of FCOPs to inhibit IL-6 secretion was more significant in NIH/3T3 than in HUVECs. Concerning MMP-3 and ICAM-1, their concentrations under physiological or pathological conditions were both higher in HUVECs than in NIH/3T3. However, in NIH/3T3, the degree of alteration in MMP-3 was greater than in HUVECs under 200µM H_2_O_2_ exposure, with a nearly 10-fold increase compared to the vehicle control. Therefore, the inhibitory effect of FCOPs on MMP-3 appears to be weak in NIH/3T3. The anti-inflammatory properties of fish collagen peptides have been reported in multiple studies [40,41,42]. In HUVECs, despite the high production of ROS, FCOPs still significantly inhibited the secretion of the above inflammatory factors. This is partially due to the high intrinsic baseline ROS level in this cell line. On the other hand, this also suggests that the anti-inflammatory properties of FCOPs are not solely attained by direct control of ROS levels. FCOPs may directly affect the inflammatory signaling pathways, release of inflammatory mediators, or regulate immune cell function. We considered a multi-subunit transcription factor called nuclear factor-κB (NF-κB), which quickly triggers the expression of genes related to inflammation, immunology, and acute phase responses [43]. The present paper found that FCOPs significantly suppressed the activation of NF-κB, while the vehicle and H_2_O_2_ control group expressed a similar amount of NF-κB with different levels of ROS. It is suggested that FCOPs inhibited inflammatory response directly through NF-κB. Furthermore, our in vivo investigation showed that 1.350 g/(kg.d) FCOP administration for 30 days significantly down-regulated the spleen relative protein expression of NF-κB in 6 Gy (1.5 Gy/min) ^60^Coγ-rays irradiated C57BL mice [19]. Additionally, eNOS exerts anti-inflammatory effects by blocking the activation of inflammatory cells and the secretion of inflammatory cytokines [44,45]. The study found that FCOPs significantly enhanced eNOS activity. Therefore, FCOPs not only interrupted the feedback loop between oxidative stress and inflammation, but also directly blocked the inflammatory response signaling pathways.

Mitochondrial function and ATP synthesis are crucial for normal cellular function. Mitochondria are the principal origin of ROS production in the body and are responsible for cellular energy production. Oxidative stress causes mitochondrial dysfunction, which is signified by changes in mitochondrial morphology and loss of function. This condition leads to a positive feedback loop of further ROS production in mitochondria [46,47]. Therefore, the present research was designed to investigate the effects of FCOPs on mitochondria for the first time. Disturbances of mitochondrial membrane potential (MMP) are a crucial hallmark of mitochondrial dysfunction. MMP deficiency can cause abnormalities in the mitochondrial electron transport chain, reduced oxygen expenditure, diminished ATP stores, and impaired energy metabolism. Our findings indicate that FCOPs can significantly enhance mitochondrial membrane potential and increase ATP synthesis in NIH/3T3 and HUVECs. Consistent with ROS levels, the degree of alteration in mitochondrial membrane potential and ATP production was dramatic in NIH/3T3 due to significant inhibition of ROS production by FCOPs. For HUVECs, FCOPs enhanced mitochondrial membrane potential to a lesser extent because of their higher ROS levels. However, ATP synthesis was significantly increased in both cell types, suggesting that FCOPs may affect metabolic pathways or ATP synthase activity. Given that the electron transport chain converts O_2_ to water and NADH to NAD^+^ during ATP production, we further examined NAD^+^ activity and NAD^+^/NADH. In the present study, we found that FCOP interventions restored NAD^+^ activity and NAD^+^/NADH, which were reduced during oxidative stress, to normal levels. NAD^+^ is an important coenzyme in energy metabolism; however, it has various functions other than that of a coenzyme. A variety of enzymes employ NAD^+^ and its metabolites as digestion substrates, linking cellular metabolism to alterations in signaling and transcriptional processes [48]. Therefore, we further explored the effects of FCOPs on the NAD^+^-involved mitochondrial biogenesis-related signaling pathway, AMPKα/NAD^+^/SIRT1/PGC-1α. We observed that FCOPs had no impact on AMPKα expression, as there were no statistical differences among groups and there were different trends in the two cell models. Therefore, we hypothesize that the observed increase in NAD^+^ activity by FCOPs may not have been mediated by AMPKα. Additionally, FCOPs did not elevate the expression of SIRT1 and even reduced its expression in HUVECs. SIRT1 is a NAD^+^-dependent coenzyme [49], and due to the significant decrease in cellular NAD^+^ levels caused by hydrogen peroxide treatment, SIRT1 cannot be activated to function normally. This may explain the higher expression of SIRT1 protein in the H_2_O_2_ control group. However, FCOPs tended to increase the expression of PGC-1α, with a consistent trend in both cell models. As a natural food ingredient, the effects of FCOPs are milder compared to those of drugs. We speculate that extending the intervention time of FCOPs and increasing the dose may lead to better outcomes. Transcriptional coactivator PGC-1α is the fundamental determinant of mitochondrial biogenesis and function [50]. It controls the expression of mitochondrial antioxidant genes, which helps prevent oxidative stress and mitochondrial malfunction [51]. Low levels of PGC-1α disrupt redox homeostasis, exacerbate inflammation, and are linked to metabolic syndrome, such as obesity, type 2 diabetes, cardiovascular diseases, and other related conditions [52]. Therefore, PGC-1α serves as a crucial link between oxidative stress and mitochondrial function, inflammation, and metabolic syndrome, making it an essential therapeutic target.

In general, FCOPs have the ability to resist oxidative stress induced by hydrogen peroxide, inhibit inflammation, protect mitochondria, and ensure normal cellular activity in two different cellular models. PGC-1α may be one of their targets. The antioxidant effects of FCOPs on the two types of cells were relatively consistent, with some variations in effectiveness. The results showed a more significant effect on NIH/3T3 cells, indicating differences in the absorption mechanisms and efficiency of FCOPs in different tissues. Therefore, it is necessary to use more stable cell models, combined with in vivo studies, to verify the implications of FCOPs on the aforementioned targets and extensively explore the effective intervention doses and times of FCOPs in different tissues.

## 5. Conclusions

This study investigated the antioxidant effects and the potential mechanisms of FCOPs at the cellular level. We have shown that FCOPs exhibited antioxidant properties in NIH/3T3 and HUVECs damaged by oxidative stress by inhibiting ROS production, enhancing antioxidant enzyme activities, promoting cell viability, inhibiting cell cycle arrest and inflammation, and protecting mitochondrial function. The molecular mechanism for this may involve the improvement of mitochondrial biogenesis through the NAD^+^/SIRT1/PGC1α signaling pathway. FCOPs exhibited superior antioxidant effects on NIH/3T3 than on HUVECs. This study complements findings related to the antioxidant effects of FCOPs and provides a basis for further in-depth studies targeting mitochondria. This study is crucial for a comprehensive understanding of the antioxidant effects of FCOPs.

## Figures and Tables

**Table 1:** The amino acid ratio of Fish Collagen Oligopeptides (FCOPs).

Amino Acid	Ratio (g/100 g)	Amino Acid	Ratio (g/100 g)
Glycine	23.67	Threonine	2.77
Glutamic Acid	11.22	Leucine	2.62
Proline	10.42	Valine	2.34
Alanine	10.24	Phenylalanine	1.90
Hydroxyproline	8.72	Isoleucine	1.30
Arginine	8.34	Methionine	1.06
Aspartic Acid	5.44	Histidine	0.75
Lysine	3.40	Tyrosine	0.32
Serine	2.83	Cystine	0.04

## Data Availability

The data presented in present paper are available on request from the corresponding author. The data are not publicly available due to privacy.

## References

[1] Sies H., Berndt C., Jones D.P. (2017). Oxidative Stress. Annu. Rev. Biochem..

[2] Sies H. (2015). Oxidative stress: A concept in redox biology and medicine. Redox Biol..

[3] Pizzino G., Irrera N., Cucinotta M., Pallio G., Mannino F., Arcoraci V., Squadrito F., Altavilla D., Bitto A. (2017). Oxidative Stress: Harms and Benefits for Human Health. Oxidative Med. Cell. Longev..

[4] Freudenthal B.D., Beard W.A., Perera L., Shock D.D., Kim T., Schlick T., Wilson S.H. (2015). Uncovering the polymerase-induced cytotoxicity of an oxidized nucleotide. Nature.

[5] Crisóstomo L., Oliveira P.F., Alves M.G. (2022). Antioxidants, Oxidative Stress, and Non-Communicable Diseases. Antioxidants.

[6] Teleanu D.M., Niculescu A.G., Lungu I.I., Radu C.I., Vladâcenco O., Roza E., Costăchescu B., Grumezescu A.M., Teleanu R.I. (2022). An Overview of Oxidative Stress, Neuroinflammation, and Neurodegenerative Diseases. Int. J. Mol. Sci..

[7] Sharifi-Rad M., Anil Kumar N.V., Zucca P., Varoni E.M., Dini L., Panzarini E., Rajkovic J., Tsouh Fokou P.V., Azzini E., Peluso I. (2020). Lifestyle, Oxidative Stress, and Antioxidants: Back and Forth in the Pathophysiology of Chronic Diseases. Front. Physiol..

[8] Senoner T., Dichtl W. (2019). Oxidative Stress in Cardiovascular Diseases: Still a Therapeutic Target?. Nutrients.

[9] López-Otín C., Blasco M.A., Partridge L., Serrano M., Kroemer G. (2023). Hallmarks of aging: An expanding universe. Cell.

[10] Liguori I., Russo G., Curcio F., Bulli G., Aran L., Della-Morte D., Gargiulo G., Testa G., Cacciatore F., Bonaduce D. (2018). Oxidative stress, aging, and diseases. Clin. Interv. Aging.

[11] Ursini F., Maiorino M., Forman H.J. (2016). Redox homeostasis: The Golden Mean of healthy living. Redox Biol..

[12] Griendling K.K., Camargo L.L., Rios F.J., Alves-Lopes R., Montezano A.C., Touyz R.M. (2021). Oxidative Stress and Hypertension. Circ. Res..

[13] Harrington J.S., Ryter S.W., Plataki M., Price D.R., Choi A.M.K. (2023). Mitochondria in health, disease, and aging. Physiol. Rev..

[14] Downer S., Berkowitz S.A., Harlan T.S., Olstad D.L., Mozaffarian D. (2020). Food is medicine: Actions to integrate food and nutrition into healthcare. BMJ.

[15] Bhat S., Coyle D.H., Trieu K., Neal B., Mozaffarian D., Marklund M., Wu J.H.Y. (2021). Healthy Food Prescription Programs and their Impact on Dietary Behavior and Cardiometabolic Risk Factors: A Systematic Review and Meta-Analysis. Adv. Nutr..

[16] Dong Y., Dai Z. (2022). Physicochemical, Structural and Antioxidant Properties of Collagens from the Swim Bladder of Four Fish Species. Mar. Drugs.

[17] Yang R., Wang J., Liu Z., Pei X., Han X., Li Y. (2011). Antioxidant effect of a marine oligopeptide preparation from chum salmon (*Oncorhynchus keta*) by enzymatic hydrolysis in radiation injured mice. Mar. Drugs.

[18] Liang J., Li Q., Lin B., Yu Y., Ding Y., Dai X., Li Y. (2014). Comparative studies of oral administration of marine collagen peptides from Chum Salmon (*Oncorhynchus keta*) pre- and post-acute ethanol intoxication in female Sprague-Dawley rats. Food Funct..

[19] Yang R., Pei X., Wang J., Zhang Z., Zhao H., Li Q., Zhao M., Li Y. (2010). Protective effect of a marine oligopeptide preparation from chum salmon (*Oncorhynchus keta*) on radiation-induced immune suppression in mice. J. Sci. Food Agric..

[20] Zhu C.F., Li G.Z., Peng H.B., Zhang F., Chen Y., Li Y. (2010). Treatment with marine collagen peptides modulates glucose and lipid metabolism in Chinese patients with type 2 diabetes mellitus. Appl. Physiol. Nutr. Metab. Physiol. Appl. Nutr. Metab..

[21] Lin B., Zhang F., Yu Y., Jiang Q., Zhang Z., Wang J., Li Y. (2012). Marine collagen peptides protect against early alcoholic liver injury in rats. Br. J. Nutr..

[22] Liang J., Pei X.R., Wang N., Zhang Z.F., Wang J.B., Li Y. (2010). Marine collagen peptides prepared from chum salmon (*Oncorhynchus keta*) skin extend the life span and inhibit spontaneous tumor incidence in Sprague-Dawley Rats. J. Med. Food.

[23] Zhai L., Xu X., Liu J., Jing C., Yang X., Zhao D., Jiang R., Sun L.W. (2021). A Novel Biochemical Study of Anti-Dermal Fibroblast Replicative Senescence Potential of Panax Notoginseng Oligosaccharides. Front. Pharmacol..

[24] Schneider V.S., Bark J.M., Winnischofer S.M.B., Dos Santos E.F., Iacomini M., Cordeiro L.M.C. (2020). Dietary fibres from guavira pomace, a co-product from fruit pulp industry: Characterization and cellular antioxidant activity. Food Res. Int..

[25] Liang B., Xiang Y., Zhang X., Wang C., Jin B., Zhao Y., Zheng F. (2020). Systematic Pharmacology and GEO Database Mining Revealed the Therapeutic Mechanism of Xuefu Zhuyu Decoration for Atherosclerosis Cardiovascular Disease. Front. Cardiovasc. Med..

[26] Cao X., Bi R., Hao J., Wang S., Huo Y., Demoz R.M., Banda R., Tian S., Xin C., Fu M. (2020). A study on the protective effects of taxifolin on human umbilical vein endothelial cells and THP-1 cells damaged by hexavalent chromium: A probable mechanism for preventing cardiovascular disease induced by heavy metals. Food Funct..

[27] Singh A. (2022). Quantifying radiation damage. Nat. Methods.

[28] Sheng Y., Qiu Y.T., Wang Y.M., Chi C.F., Wang B. (2022). Novel Antioxidant Collagen Peptides of Siberian Sturgeon (*Acipenserbaerii*) Cartilages: The Preparation, Characterization, and Cytoprotection of H_2_O_2_-Damaged Human Umbilical Vein Endothelial Cells (HUVECs). Mar. Drugs.

[29] Ren Y., Wu H., Chi Y., Deng R., He Q. (2020). Structural characterization, erythrocyte protection, and antifatigue effect of antioxidant collagen peptides from tilapia (*Oreochromis nilotica* L.) skin. Food Funct..

[30] Chen Y.P., Liang C.H., Wu H.T., Pang H.Y., Chen C., Wang G.H., Chan L.P. (2018). Antioxidant and anti-inflammatory capacities of collagen peptides from milkfish (*Chanos chanos*) scales. J. Food Sci. Technol..

[31] Xia E., Zhu X., Gao X., Ni J., Guo H. (2021). Antiaging Potential of Peptides from Underused Marine Bioresources. Mar. Drugs.

[32] Chen S., Yang Q., Chen X., Tian Y., Liu Z., Wang S. (2020). Bioactive peptides derived from crimson snapper and in vivo anti-aging effects on fat diet-induced high fat Drosophila melanogaster. Food Funct..

[33] Sies H., Belousov V.V., Chandel N.S., Davies M.J., Jones D.P., Mann G.E., Murphy M.P., Yamamoto M., Winterbourn C. (2022). Defining roles of specific reactive oxygen species (ROS) in cell biology and physiology. Nat. Rev. Mol. Cell Biol..

[34] Heo A.J., Kim S.B., Ji C.H., Han D., Lee S.J., Lee S.H., Lee M.J., Lee J.S., Ciechanover A., Kim B.Y. (2021). The *N*-terminal cysteine is a dual sensor of oxygen and oxidative stress. Proc. Natl. Acad. Sci. USA.

[35] Wang L.H., Gu Z.W., Li J., Yang W.Q., Li Y.L., Qi D.M., Wang D.Y., Jiang H.Q. (2023). Isorhynchophylline inhibits inflammatory responses in endothelial cells and macrophages through the NF-κB/NLRP3 signaling pathway. BMC Complement. Med. Ther..

[36] Lee Y., Im E. (2021). Regulation of miRNAs by Natural Antioxidants in Cardiovascular Diseases: Focus on SIRT1 and eNOS. Antioxidants.

[37] Inbaraj B.S., Lai Y.W., Chen B.H. (2024). A comparative study on inhibition of lung cancer cells by nanoemulsion, nanoliposome, nanogold and their folic acid conjugates prepared with collagen peptides from Taiwan tilapia skin. Int. J. Biol. Macromol..

[38] Gaspardi A.L.A., da Silva D.C., Ponte L.G.S., Galland F., da Silva V.S.N., Simabuco F.M., Bezerra R.M.N., Pacheco M.T.B. (2022). In vitro inhibition of glucose gastro-intestinal enzymes and antioxidant activity of hydrolyzed collagen peptides from different species. J. Food Biochem..

[39] Haghi Aminjan H., Abtahi S.R., Hazrati E., Chamanara M., Jalili M., Paknejad B. (2019). Targeting of oxidative stress and inflammation through ROS/NF-kappaB pathway in phosphine-induced hepatotoxicity mitigation. Life Sci..

[40] Rahabi M., Salon M., Bruno-Bonnet C., Prat M., Jacquemin G., Benmoussa K., Alaeddine M., Parny M., Bernad J., Bertrand B. (2022). Bioactive fish collagen peptides weaken intestinal inflammation by orienting colonic macrophages phenotype through mannose receptor activation. Eur. J. Nutr..

[41] Woo M., Seol B.G., Kang K.H., Choi Y.H., Cho E.J., Noh J.S. (2020). Effects of collagen peptides from skate (*Raja kenojei*) skin on improvements of the insulin signaling pathway via attenuation of oxidative stress and inflammation. Food Funct..

[42] Subhan F., Kang H.Y., Lim Y., Ikram M., Baek S.Y., Jin S., Jeong Y.H., Kwak J.Y., Yoon S. (2017). Fish Scale Collagen Peptides Protect against CoCl_2_/TNF-α-Induced Cytotoxicity and Inflammation via Inhibition of ROS, MAPK, and NF-κB Pathways in HaCaT Cells. Oxidative Med. Cell. Longev..

[43] Liu P., Li Y., Wang W., Bai Y., Jia H., Yuan Z., Yang Z. (2022). Role and mechanisms of the NF-κB signaling pathway in various developmental processes. Biomed. Pharmacother..

[44] Chook C.Y.B., Cheung Y.M., Ma K.Y., Leung F.P., Zhu H., Niu Q.J., Wong W.T., Chen Z.Y. (2023). Physiological concentration of protocatechuic acid directly protects vascular endothelial function against inflammation in diabetes through Akt/eNOS pathway. Front. Nutr..

[45] Ku C.W., Ho T.J., Huang C.Y., Chu P.M., Ou H.C., Hsieh P.L. (2021). Cordycepin Attenuates Palmitic Acid-Induced Inflammation and Apoptosis of Vascular Endothelial Cells through Mediating PI3K/Akt/eNOS Signaling Pathway. Am. J. Chin. Med..

[46] Kim S.H., Kim H. (2018). Inhibitory Effect of Astaxanthin on Oxidative Stress-Induced Mitochondrial Dysfunction-A Mini-Review. Nutrients.

[47] Chen Y., Zhou Z., Min W. (2018). Mitochondria, Oxidative Stress and Innate Immunity. Front. Physiol..

[48] Cantó C., Menzies K.J., Auwerx J. (2015). NAD^+^ Metabolism and the Control of Energy Homeostasis: A Balancing Act between Mitochondria and the Nucleus. Cell Metab..

[49] Gao W., Li R., Ye M., Zhang L., Zheng J., Yang Y., Wei X., Zhao Q. (2022). The circadian clock has roles in mesenchymal stem cell fate decision. Stem Cell Res. Ther..

[50] Abu Shelbayeh O., Arroum T., Morris S., Busch K.B. (2023). PGC-1α Is a Master Regulator of Mitochondrial Lifecycle and ROS Stress Response. Antioxidants.

[51] Jannig P.R., Dumesic P.A., Spiegelman B.M., Ruas J.L. (2022). SnapShot: Regulation and biology of PGC-1α. Cell.

[52] Rius-Pérez S., Torres-Cuevas I., Millán I., Ortega Á.L., Pérez S. (2020). PGC-1α, Inflammation, and Oxidative Stress: An Integrative View in Metabolism. Oxidative Med. Cell. Longev..

